# The Diversity of Human Dirofilariasis in Western Sri Lanka

**DOI:** 10.1155/2019/9209240

**Published:** 2019-04-18

**Authors:** T. G. A. N. Chandrasena, R. Premaratna, C. H. Mallawaarachchi, N. K. Gunawardena, P. A D. H. N. Gunathilaka, W. Y. Abeyewickrama, N. R. de Silva

**Affiliations:** ^1^Department of Parasitology, Faculty of Medicine, University of Kelaniya, Sri Lanka; ^2^Department of Medicine, Faculty of Medicine, University of Kelaniya, Sri Lanka; ^3^Postgraduate Institute of Medicine, Ministry of Health, Sri Lanka; ^4^Colombo South Teaching Hospital, Ministry of Health, Sri Lanka

## Abstract

**Background:**

Human dirofilariasis is an emerging zoonosis in many countries. Dirofilariasis caused by* Dirofilaria repens* may present with diverse clinical manifestations in humans due to aberrant localization of worm lesions causing diagnostic dilemmas. The aim of this retrospective study was to describe and update the demography and clinical spectrum of human dirofilariasis in western Sri Lanka. Nematode or nematode fragments isolated from excision biopsies that were confirmed as* D. repens* at the Department of Parasitology, Faculty of Medicine, University of Kelaniya, Sri Lanka, between 2012 and 2018 were included. Data on age, gender, and clinical details were obtained from case files. Identity of worms was established by morphometry and cuticle characteristics on wet-mount preparation. Specimens from unusual case presentations were further analyzed by PCR with specific primers for internal transcribed spacer region 2 (ITS2) of the ribosomal DNA.

**Results:**

Sixteen nematode specimens isolated from subconjunctiva (n=2), subcutaneous (n=13) and intramuscular (n=1) locations were identified as* D. repens *by morphometry (average length 11.5 cm) and the characteristic longitudinal striations on cuticle visualized by microscopy. The age distribution of cases ranged from 1 to 65 years with a mean of 21.5. Females were more frequently affected (n=10, 62.5%) and worm locations were commonest in the orbital region (5/16) and scrotum (3/16). Imaging techniques were of use in detecting infections in deeper tissue levels. PCR analysis of DNA extracted from a worm in an intramuscular granuloma of the temporal region elicited the expected band at 484bp for* D. repens*.

**Conclusions:**

Human dirofilariasis is on an upward trend in incidence. Imaging techniques were of use in clinical diagnosis and molecular speciation in establishing the species identity in unusual case presentations. We suggest a more conservative approach in the management of human dirofilariasis and recommend a one health approach for control.

## 1. Background

Dirofilariasis is a group of zoonotic filarial infections mainly of dogs (natural host), cats, and other carnivores. Two species are mostly of relevance with regard to human infections,* Dirofilaria (Nochtiella) repens *and* D. immitis *[1, 2]. In the natural definitive hosts (dogs), the adult worms of* D. repens* are mainly found in the subcutaneous tissues and D.* immitis* infections are related to the presence of adult worms in the pulmonary artery and right ventricle of hearts while microfilaria of both species are usually found in the blood stream.

The life cycle of* Dirofilaria *species is similar to that of other filarial parasites, with a definitive vertebrate host and a vector. Both* D. immitis* and* D. repens* demonstrate low vertebrate host specificity as they are able to infect numerous mammalian species. They are best adapted to canines but cats also can act as reservoirs of* D. (N.) repens* [2]. Humans are regarded as less suitable hosts as parasites usually do not mature sufficiently to produce microfilaraemia. But occasional reports of subcutaneous dirofilariasis with circulating microfilaraemia in humans contradict the commonly accepted belief that humans are dead-end hosts [2, 3].

Vectors comprise female mosquitoes of the Culicidae family [4]. Both* D. repens* and* D. immitis* themselves are hosts to essential symbiotic bacteria of the genus* Wolbachia* that has been shown to play an important role in filarial biology and host-parasite interactions [5].

Of the two species,* D. immitis* is more widespread in distribution, involving tropical and temperate regions of the Old World as well as the New World [6] while* D. repens* is limited to Asia, Africa, and Europe [2, 7]. At present dirofilariasis is regarded as an emerging zoonosis in many countries [2, 7–9].

Human infections with* D. repens* commonly affect the subcutaneous or subconjunctival tissues while* D. immitis* is associated with pulmonary dirofilariasis [10]. However,* D. repens* may infect a variety of anatomical sites including the peritoneum, omentum, lungs, and soft tissues (breasts and muscles) [11–14]. Transient inflammatory swellings or nodules signal the presence of worms while in more sensitive tissues such as conjunctiva an acute inflammatory response occurs prompting immediate medical attention [10].

Subcutaneous and ocular dirofilariasis caused by* D. repens* is one of the most frequently detected parasitic zoonosis in Sri Lanka [15, 16]. On the other hand, cardiopulmonary dirofilariasis caused by* D. immitis* has not been described in Sri Lanka to date. A global review of cases from 1995 to 2000 has documented the second largest collection of subcutaneous and ocular* D. repens* infections from Sri Lanka [11]. Subsequently a series of intraoral infections were reported in 2003 [17] and 2015 [18] and 30 cases of ocular infections were reported from 2006 to 2014 from the central province of Sri Lanka [16]. Moreover, the prevalence of canine and feline* D. repens* infections continues to be high in western Sri Lanka [19, 20].

Differentiation of worm granulomata from other causes of nodules is an important aspect in the clinical management of human dirofilariasis. Thus surgery is recommended, mostly to exclude a malignant origin of a nodule or for removal of worms in ocular locations. Chemotherapy is usually not practiced [21]. Although most infections cause minor disease, serious consequences ranging between impaired or loss of vision and meningoencephalitis have occurred [2, 12].

The diversity of human* D. repens* disease manifestations has often caused diagnostic dilemmas [22]. This study provides an update on the epidemiology and diversity of disease manifestations of* D. repens* infections in western Sri Lanka.

## 2. Methods

This retrospective case analysis was conducted from 2012 to 2018 in the Department of Parasitology, Faculty of Medicine, University of Kelaniya, Sri Lanka. All excision biopsy specimens from which the entire nematode or nematode fragments were extracted were included. Worm identity was established by morphometry and the presence of longitudinal striations on cuticle visualized in wet-mount preparations by microscopy [10, 23]. Molecular speciation of extracted worm specimens was performed on an unusual case presentation of an intramuscular worm granuloma (case No. 16 in Table 1).

Extraction of genomic DNA from worm fragments was performed using the Qiagen minikit (Qiagen DY14, Hilden, Germany) as per manufacturer's instructions. The extracted DNA was analyzed by PCR using pan-filarial primers (DIDR-F1 5'-AGT GCG AAT TGC AGA CGC ATT GAG-3' and DIDR-R1 5'-AGC GGG TAA TCA CGA CTG AGT TGA-3') specific for the internal transcribed spacer region 2 (ITS2) of the ribosomal DNA [24]. Electrophoresis of the PCR products was done on 2% agarose gel.

The demographic and clinical data that was retrieved from case files or referral letters or by direct contact with patients were analyzed. Written informed consent was obtained to publish clinical, laboratory, and imaging data of relevant patients (cases: 13 and 16, Table 1).

## 3. Results

**Figure 1:**
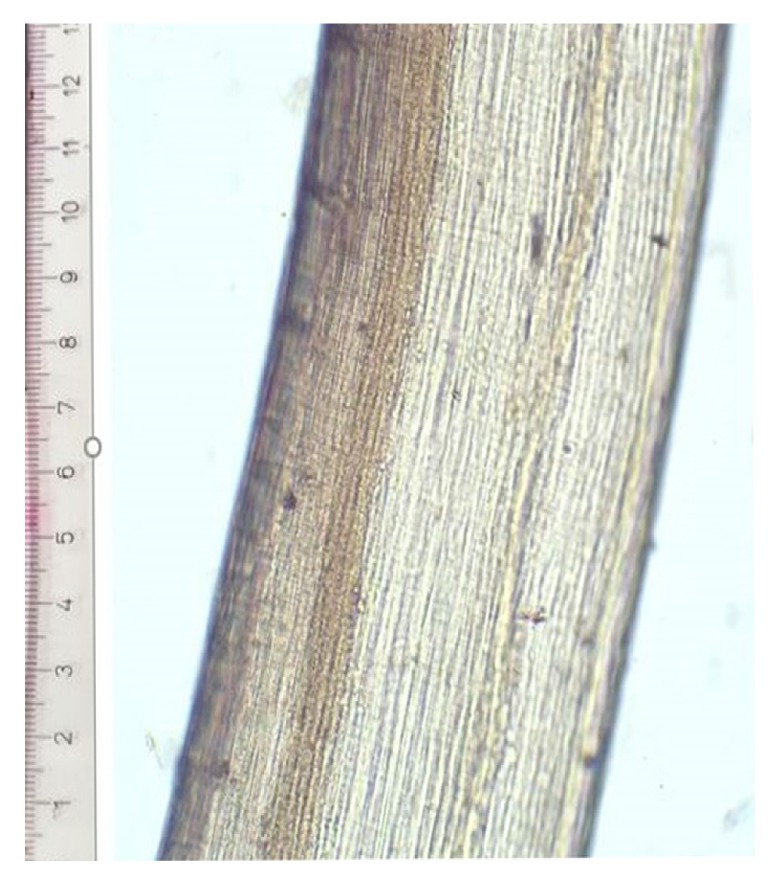
*The cuticle of D. repens as visualized by light microscopy.* The longitudinal striations on the cuticle of* D. repens* (x10).

**Figure 2:**
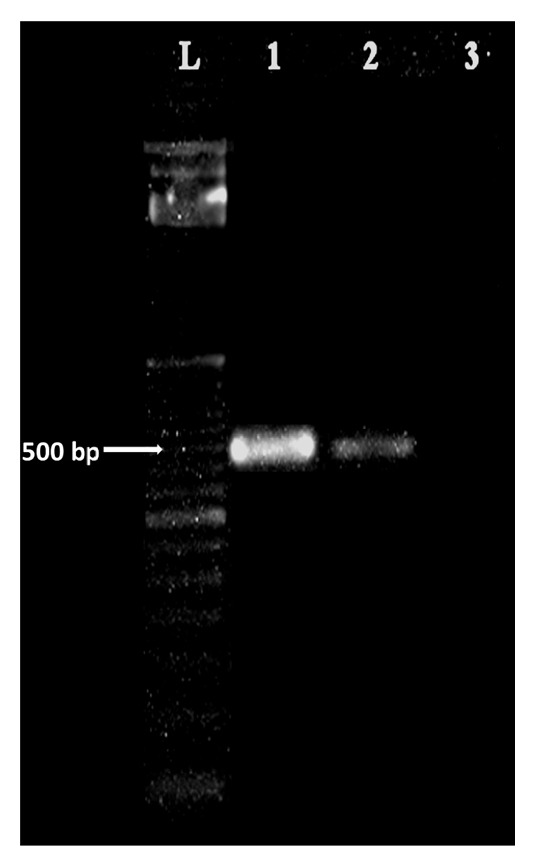
*Gel-image of PCR amplified products with pan-filarial primers, DIDR-F1 5' and DIDR-R1 5'.* Lanes L -100bp marker, 1-* D. repens* positive control, 2- case no. 16, and 3- negative control.

A total of 16 case referrals were confirmed as* D. repens* infection. The age distribution of cases varied from 1 to 65 years with a mean of 21.5. Nematode specimens were extracted from subconjunctival (n=2), subcutaneous (n=13), and intramuscular tissues (n=1). Worms were mostly extracted from the orbital region (6/16) and scrotum (3/16) as shown in Table 1. All worms were identified as* D. repens *by morphometry (average length 11.5 cm) and the characteristic longitudinal cuticular striations (see Figure 1). Molecular speciation of the worm specimen extracted from the left superficial temporalis muscle (case No. 16) by PCR elicited the expected band at 484bp confirming the identity of the worm as* D. repens* (Figure 2).

Brief descriptions of the two aberrant case presentations are included to make clinicians aware of such manifestations.

### 3.1. Migrating Worm in the Eye (Case No. 13 in Table 1) 

A 34-year-old female resident from Seeduwa (a semiurban area located 25 km north of Colombo, along the western coastal belt of Sri Lanka) presented to the ophthalmology clinic in 2016 with a history of itchiness and sensation of a foreign body in the left eye for several hours followed by sighting of a worm migrating across the eye.

**Figure 3:**
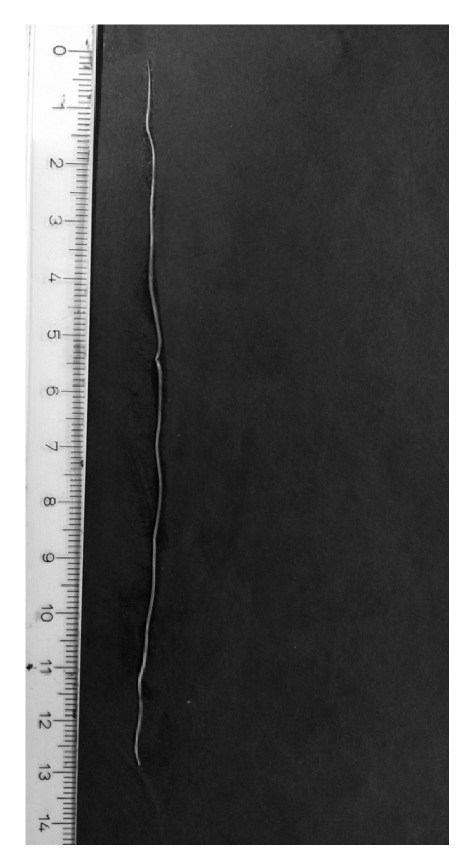
*An adult D. repens worm.* The worm extracted from the abdominal lesion in case no. 13.

At the clinic the worm was visualized by slit lamp examination but extraction failed as the worm migrated to the periorbital tissues. The following day, the patient presented to the Department of Parasitology with periophthalmitis (periorbital swelling, intermittent tearing, and red eye) for which a course of doxycycline (anti-*Wolbachia *agent) was administered combined with steroids. The ophthalmitis subsided but she experienced transient itchiness over the face and chest area. On the fifth day of treatment, an adult* D. repens* worm emerged from a “pimple” like lesion on the anterior abdominal wall accidently punctured by the patient herself (Figure 3).

### 3.2. Intramuscular Dirofilariasis in the Temporal Region Presenting as a Soft Tissue Mass (Case No.16)

A 36-year-old female medical officer residing in Colombo presented in 2018 with trismus, deviation of the mouth to a side, and mild swelling over the left temporomandibular joint (LTMJ). The swelling worsened following a molar extraction (incidental to the main pathology) and administration of intravenous antibiotic and amoxicillin/clavulanate resulted in its remission leaving a localized nodule 3cm in size over the LTMJ which was accompanied by systemic symptoms of giddiness, nausea, vomiting, and severe headache.

**Figure 4:**
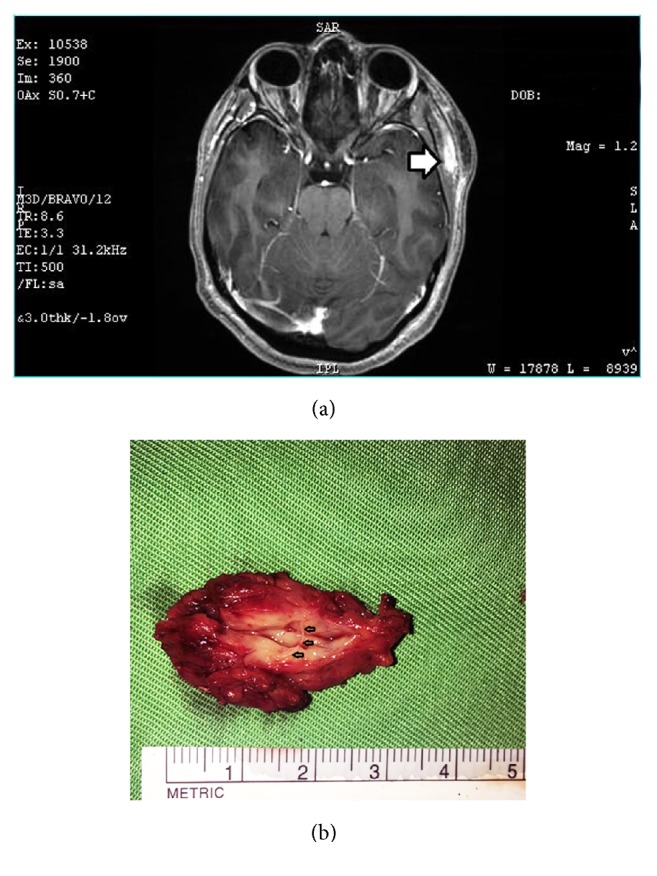
*(a) MRI scan image of the brain of case 16.* The scan image shows the contrast-enhancing tubular structure in the left temporal region with involvement of the superficial temporalis muscle (thick arrow head).* (b) Gross aspect of the excised worm granuloma in case 16.* Worm fragments (arrowheads) are visualized within the dissected specimen.

A contrast-enhanced MRI scan of the head revealed the presence of a contrast-enhancing tubular tortuous structure with a large area of oedema in the surrounding soft tissues, suggestive of inflammation (see Figure 4(a)). The imaging findings together with a mild peripheral blood eosinophilia (5.9%, reference range 0.5-5) raised the possibility of an underlying parasitic granuloma. Ultrasonography of the region also revealed the presence of a tubular hyperechoic lesion (2.2 cm x 0.7 cm x 0.5 cm) within the left superficial temporalis muscle. Lack of movements of the tubular structure suggested a dead worm. Administration of antifilarials for a week {diethylcarbamazine citrate (DEC) 6mg/kg, albendazole 200mg and doxycycline 100mg twice daily} resulted in minimal clinical improvement. A dose of intravenous steroid and dexamethasone provided partial remission but symptoms recurred with cessation of therapy.

Excision was done under general anaesthesia with an incision placed 2cm posterior to the hairline. A sausage shaped lump with ill-defined margins, 3 cm x 1.5 cm x 0.7 cm in size, was removed by incising the superficial temporalis muscle (Figure 4(b)). Whitish worm fragments 2-3cm in size were noted within the dissected specimen. Recovery was uneventful with a small residual nodule on the LTMJ.

## 4. Discussion

A combination of conventional microscopy and molecular techniques was applied in the confirmation of* D. repens* infections at a reference laboratory situated in western Sri Lanka. The frequency of* D. repens* isolations in the present series appears to follow an upward global trend.

Similar to past reviews, subcutaneous dirofilariasis commonly manifested as solitary nodules and less frequently as migratory swellings in this series (Table 1) [11]. Females were more frequently affected as reported in the past (10/16; 62.5%) [11, 16]. In accordance with the world literature the majority of lesions (10/16; 62.5%) were located on the upper half of the body [11, 16], with the face being the commonest site affected (7/16; 43.75%). In the lower half of the body, scrotum was a favored location (3/16; 18.8%) as reported in the past by Dissanaike et al. [15]. The aggregation of lesions on the upper half of the body has been explained by the tendency for parasites to locate near points of mosquito bites [1] which are higher on exposed regions such as face, neck, and upper limbs. The reason for favoring scrotal tissues is unknown and appears to be unique to Sri Lankan cases [15].

The deeper tissue level infections have been attributed to the tendency for adult/immature worms to migrate within soft tissues until restricted by host defenses at its final anatomical location [1]. The common age groups affected in the present series were the 1-10 (5/16; 31.2%) and 11-20 (6/16; 37.5%) years' age groups, which was somewhat similar to that reported by Dissanaike et al. in the past (less than 9 years) [15] but was much younger than the common age group of 40–49 years, reported by Iddawela et al. for ocular dirofilariasis in the cooler climes of the central province of Sri Lanka [16] and those reported from the European region (40-49 years) [11]. Perhaps the practice of leaving children minimally clothed because of the warm and humid weather in the western region may have exposed them more to mosquito bites and infection.

Worldwide, seven cases of dirofilariasis with localization in the temporal region have been described up to 2017 [14]; only two of them were in intramuscular locations [11, 14]. Thus this case series documents the third case of intramuscular dirofilariasis of the temporal region caused by* D. repens*. Therefore, it is important to consider dirofilariasis in the differential diagnosis of soft tissue masses located at deeper levels. Ocular dirofilariasis in the present series involved the infraorbital and subconjunctival tissues but none were in intraocular tissues, which is documented as uncommon [25].

Surgical extraction of worms from superficial tissues or from the conjunctiva is a simple procedure but intervention is more complex in the case of internal locations especially in the head and neck regions. With the advent of advanced imaging techniques which enable differentiation of worm granulomata from other nodules/masses with a high degree of accuracy [26] and the discovery of an important therapeutic target (essential symbiont,* Wolbachia*) [27, 28], a more conservative management approach may be feasible. It is documented that administration of DEC does not kill the worms, but arrests their migration and causing them to settle at one spot, after which they emerged from lesions spontaneously or by application of pressure on lesions (squeezing) [15]. A similar outcome was observed with doxycycline treatment in case no. 13 of the present series. The outcome of chemotherapy in the intramuscular infection (case no. 16) differed. It is possible that the death of the worm in this case was mediated by the host's immune response [10]. This theory is supported by the ultrasonographic findings which suggested a dead worm. The secondary inflammatory response initiated by the lipopolysaccharide-like activity of the symbiotic* Wolbachia* bacteria, released from the tissues of the dead worm [29–31], may have initiated the pathological manifestations. Thus, administration of filaricides (DEC and albendazole) and* Wolbachia *clearing agents (doxycycline) provided minimal improvement at that stage but steroid therapy provided remission of symptoms as in the case reported by Poppert et al. [12]. With the rising incidence it would be timely to explore the place of chemotherapy that may result in regression of worm granulomata without surgical intervention.

This retrospective survey was based on referrals of worms or worm fragments to a single center in western Sri Lanka and thereby is limited in its ability to reflect the dirofilariasis situation in the entire country. Even regionally it portrays only the “tip of the iceberg” as histological referrals were not included in the current series due to difficulties encountered in data retrieval. The recent spurt of infections may also set a trend for by-passing referrals for parasitological diagnosis. Thus, many cases may remain undiagnosed and go unreported. Another drawback in this case series was the application of molecular speciation only on two specimens. However, worm identification on gross features is reliable provided the intact worm is recovered from tissues [10].

Currently human dirofilariasis is a low priority infection with scant attention being focused on control, perhaps because most infections cause only minor disease as in the current case series. However, the psychological impact of infection during the acute phase was immense because the idea of harboring migrant worms was very distressing. Ocular infections were particularly worrying due to potential visual complications.

Recent environmental modifications in the western province of Sri Lanka such as creation of water bodies within city limits as part of storm water management projects may have increased vector abundance and development of landfill sites in the suburbs for human habitation increased their exposure to infection. The zoonotic reservoir of infection [19, 20] and low levels of public awareness in the western province of Sri Lanka (18.5%) [19] are other factors contributing towards the rise in infections. Controlling canine and feline dirofilariasis by chemotherapy/chemoprophylaxis as well as reduction of vector populations are mandatory measures in combating the rise of human dirofilariasis. These two strategies may also provide a bonus effect in arresting the spread of* Brugia malayi* (subperiodic) of probable zoonotic origin that has emerged in Sri Lanka during the postelimination era of lymphatic filariasis [32].

## 5. Conclusions

Human dirofilariasis continues on an upward trend in incidence in Sri Lanka with diverse manifestations which included a case of* D. repens* infection in the temporal region with intramuscular localization of the worm. Imaging techniques were of use in clinical diagnosis and molecular speciation in establishing the species identity in such unusual case presentations. We suggest a more conservative approach in the management of human dirofilariasis and recommend close collaboration with medical doctors and veterinarians for control of human dirofilariasis.

## Figures and Tables

**Table 1:** Demography and clinical details of confirmed* D. repens* infections (2012-2018).

Case no.	Year	Worm Location	Symptoms	Age (year)	Gender M^a^/F^b^	Worm species & comments
1	2012	Encysted	Redness, tearing of left eye	65	F	*D.repens* Live
		Conjunctiva				
2	2013	Subcutaneous,	Lump on the right buttock	8	M	*D.repens* Dead
		right buttock				
3	2013	Conjunctiva	Redness, itching of left eye	18	F	*D.repens *Dead
4	2013	Subcutaneous	Cystic lump	20	F	*D.repens *Dead
		Infraorbital				
5	2014	Subcutaneous, left index finger	Cystic lump	14	F	*D.repens* Dead encysted in a sac
6	2015	Subcutaneous,	Lump	20	F	*D.repens *Dead
		Infraorbital				
7	2015	Subcutaneous, scrotum	Lump	1	M	*D.repens *Live
8	2016	Subcutaneous,	Lump	46	F	*D.repens *Dead
		Infraorbital				
9	2016	Subcutaneous,	Cystic lump	2	F	*D.repens *Live
		left forearm				
10	2016	Subcutaneous,	Lump	16	M	*D.repens *live Female
		Scrotum				
11	2016	Subcutaneous,	Lump	46	M	*D.repens *Live
		Scrotum				
12	2016	Subcutaneous	Lump	2	F	*D.repens *Dead
		Abdominal wall				
13	2016	Ocular and subcutaneous	Migratory worm	34	F	*D.repens *Live
		Abdominal wall				
14	2017	Subcutaneous	Lump	14	M	*D.repens *Dead
		Thigh				
15	2018	Subcutaneous	Cystic lump	2	M	*D.repens* Dead encysted in an abscess
		Neck				
16	2018	Intramuscular,	Inflammatory mass temporomandibular joint	36	F	*D. repens* fragmented worm
		Superficial temporalis muscle				

a, Male.

b, Female.

## Data Availability

The data used to support the findings of this study are included within the article.

## Abbreviations

ITS: Internal transcribed spacer region
PCR: Polymerase chain reaction
DNA: Deoxyribonucleic acid
LTMJ: Left temporomandibular joint
DEC: Diethylcarbamazine citrate.
